# Benign skin tumors in older persons: a population-based study

**DOI:** 10.1186/s12877-025-05881-1

**Published:** 2025-04-05

**Authors:** Nelli Leskelä, Laura Huilaja, Jari Jokelainen, Suvi-Päivikki Sinikumpu

**Affiliations:** 1 Faculty of Medicine, University of Oulu, Oulu, Finland; 2 Department of Dermatology and Medical Research Center, Oulu University Hospital, P.B.20, Oulu, FIN-90029 OYS Finland; 3 Research Unit of Clinical Medicine, University of Oulu, Oulu, Finland; 4 Northern Finland Birth Cohorts, Arctic Biobank, and Infrastructure for Population Studies, Faculty of Medicine, University of Oulu, Oulu, Finland

**Keywords:** Benign skin tumor, Seborrheic keratosis, Lentigo solaris, Cherry angiomas, Melanocytic nevi, Total body skin examination, Etiology

## Abstract

**Background:**

Benign skin tumors become more common with advanced age. However, little is known about the etiology of these common lesions. Our objectives were to evaluate the risk factors for seborrheic keratosis, lentigo solaris, cherry angiomas and melanocytic nevi in an elderly population. Our candidate predisposing factors were sex, age, Fitzpatrick skin type, history of outdoor work, life style factors and anthropometric measurements and laboratory tests.

**Methods:**

In this retrospective cross-sectional study of a large, well-documented cohort, a total body skin examination (TBSE) was performed by dermatologists. The information gathered was augmented with self-reported data. The associations between benign skin tumors and the risk factors analysed with the Chi square test, Fischer exact test and analysis of variance as appropriate.

**Results:**

The study included 552 participants aged between 70 and 93 years. According to the TBSE, benign skin tumors were present in up to 78.7% in certain subsets of participants. Seborrheic keratosis was the most common lesion type, and 15.6% of all cases had > 50 lesions. Seborrheic keratosis were more common in males (*p* < 0.05), while lentigo solaris and cherry angiomas were more common in females (*p* < 0.05). A history of outdoor working associated with higher number of lentigo solaris and seborrheic keratosis lesions (*p* < 0.05). There was an association between lower glycated haemoglobin levels and the presence of multiple cherry angiomas (*p* < 0.05). Female subjects with multiple cherry angiomas had higher levels of high-density cholesterol and lower triglyceride values than in those with fewer cherry angiomas (*p* < 0.05 for both). In males, lower mean haemoglobin levels were associated with multiple cherry angiomas (*p* < 0.05).

**Conclusions:**

We found sex differences and several new possible etiological factors behind benign skin tumors which, despite being common, remain poorly characterized.

**Supplementary Information:**

The online version contains supplementary material available at 10.1186/s12877-025-05881-1.

## Background

Seborrheic keratosis is the most common benign skin tumor type, followed by lentigo solaris and cherry angiomas [1–4]. All become markedly more common with advanced patient age [4–6]. By contrast, benign melanocytic nevi are more common in younger adults than in older persons [7, 8].

**Fig. 1:**
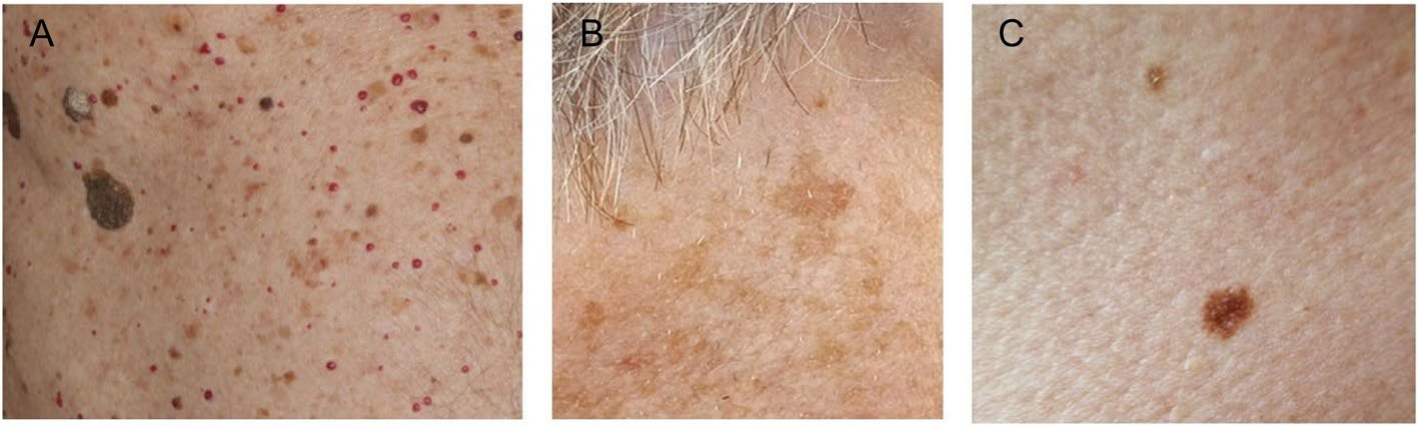
Clinical presentations of benign skin tumors. **A** Multiple seborrheic keratosis (brown tumors) and multiple cherry angiomas (red tumors); **B** Lentigo solaris; **C** Melanocytic nevus. Photos: Archives of the Department of Dermatology, Oulu University Hospital

Such tumors are one of the most common reasons for a primary care physician visit [9]. This is especially true of older persons since their skin symptoms often cause concern while they resemble those of malign tumors, and because the patients do not know why they appear. Aside from their prevalence among the elderly, the etiology of benign skin tumors has been the subject of relatively little research. According to a few existing reports, age is the main risk factor for seborrheic keratosis and cherry angiomas [10, 11]. Exposure to ultra-violet (UV) radiation can increase the incidence of seborrheic keratosis, melanocytic nevi and lentigo solaris [12–15]. There is also some evidence for a relationship between insulin resistance and seborrheic keratosis, [16] and the appearance of cherry angiomas has been linked to disturbances in patients’ lipid profiles; [17] hormonal changes may have an effect on the onset of cherry angiomas [17]. Younger individuals with fair skin types carry a higher risk of melanocytic naevi, although data are lacking in older people [18, 19]. Example phenotypes of all four of these conditions are seen in Fig. 1.

The aim of this study was to identify possible factors associated with, and predisposing for, benign skin tumors in an older population. More precisely, we aimed to study the association of sex, itching, lifestyle factors, anthropometric measurements and laboratory parameters with seborrheic keratosis, cherry angiomas, lentigo solaris and melanocytic nevi in adults aged 70 and older belonging to the Northern Finland Birth Cohort 1966 (NFBC1966) Parent Study.

## Methods

### Setting and participants

This was a cross-sectional study of Finnish adults aged from 70 to 93 years, conducted as part of the Northern Finland Birth Cohort (NFBC1966) Parents Study. The NFBC1966 is an epidemiological and longitudinal research program, based in the two northernmost provinces in Finland (Oulu and Lapland). The data set comprises diverse information on the offspring of the mothers whose expected delivery date fell between January 1 and December 31, 1966 [20, 21]. Between 2018 and 2019 the living parents of the subjects in the study were invited to respond to a health questionnaire and to participate in a comprehensive health study including a total body skin examination (TBSE) performed by a dermatologist capable of performing dermoscopy [7].

Subjects’ skin type, and all skin tumors and skin diseases found in the TBSE were recorded. The study participants were classified into four groups according to their skin type by using the modified Fitzpatrick’s criteria as follows: skin type I “skin always burns”, type II “skin burns often”, type III “skin burns occasionally” and type IV “skin never burns” [22]. In this study, “light skin type” refers to types I and II and “dark skin type” to types III and IV. The dermatologist recorded the total number of skin tumor sites for each patient and for study purposes, the presence of > 50 sites is referred to as “multiple” tumors. [7] Questionnaires were used to gather information about patients’ ‘outdoor history’ (“Do you have a history of outdoor working?”), their number of offspring, itching of the skin (“Do you have disturbing itching of the skin?”) and smoking status (“Do you currently smoke/Have you ever smoked?”). Metabolic parameters were comprehensively studied in all subjects, and these methods are described in detail in Appendix S1.

### Statistical analyses

The overall prevalence of benign skin tumors was calculated. The Chi-Square test, or Fisher's exact test when appropriate, was used to test difference in categorical variables and analysis of variance (ANOVA) for continuous variables. Statistical analyses were conducted using the R software package version 4.0.2 (https://cran.rstudio.com) and a *p*-value < 0.05 was considered statistically significant.

### Ethical approval

The Ethical Committee of the Northern Ostrobothnia Hospital District approved the study (115/2012), which was performed according to the principles of the 1983 Declaration of Helsinki. Informed written consent to participate was obtained from all study participants. The data were handled at the group level only, personal information being replaced by identification codes resulting in complete anonymity.

## Results

A total of 12,027 parents of the NFBC1966 were sent the health questionnaire. Of those, 5,559 (46.2%) responded. All the responders living in Oulu area (*n* = 1,239) were asked to participate in the clinical examination, and 552 participants (44.6%) took part. Evaluable data were available for 346 female and 205 male participants. Table 1 shows the prevalence of each of the selected study tumor types, and their associations with the selected risk factors.

**Table 1:** Benign skin tumors and their associations with the selected study candidate risk factors

	**Seborrheic keratosis**				**Lentigo solaris**				**Cherry angiomas**				**Melanocytic nevi**			
	**None**	**1–50**	** > 50**	***p* value**	**None**	**1–50**	** > 50**	***p* value**	**None**	**1–50**	** > 50**	***p* value**	**None**	**1–50**	** > 50**	***p* value**
N*	117	347	86		164	353	34		201	323	27		278	253	20	
**Sex**				0.008				< 0.001				0.009				0.951
- Female	81 (23.4)	223 (64.5)	42 (12.1)		75 (21.7)	247 (71.4)	24 (6.9)		114 (32.9)	209 (60.4)	23 (6.6)		173 (50.0)	160 (46.2)	13 (3.8)	
- Male	36 (17.6)	124 (60.8)	44 (21.6)		89 (43.4)	106 (51.7)	10 (4.9)		87 (42.4)	114 (55.6)	4 (2.0)		105 (51.2)	93 (45.4)	7 (3.4)	
**Fitzpatrick skin type**				0.126				0.265				0.300				0.007
- I	4 (23.5)	11 (64.7)	2 (11.8)		3 (17.6)	14 (82.4)	0 (0.0)		7 (41.2)	10 (58.8)	0 (0.0)		13 (76.5)	4 (23.5)	0 (0.0)	
- II	46 (26.1)	112 (63.6)	18 (10.2)		64 (36.4)	101 (57.4)	11 (6.2)		53 (30.1)	114 (64.8)	9 (5.1)		83 (47.2)	85 (48.3)	8 (4.5)	
- III	42 (17.1)	157 (63.8)	47 (19.1)		67 (27.2)	163 (66.3)	16 (6.5)		90 (36.6)	143 (58.1)	13 (5.3)		117 (47.6)	125 (50.8)	4 (1.6)	
- IV	25 (22.9)	65 (59.6)	19 (17.4)		30 (27.3)	73 (66.4)	7 (6.4)		49 (44.5)	56 (50.9)	5 (4.5)		63 (57.3)	39 (35.5)	8 (7.3)	
**History of outdoor working**				0.006				0.065				0.231				0.336
- No	105 (22.2)	304 (64.3)	64 (13.5)		144 (30.4)	306 (64.6)	24 (5.1)		174 (36.7)	274 (57.8)	26 (5.5)		241 (50.8)	218 (46.0)	15 (3.2)	
- Yes	12 (15.8)	43 (56.6)	21 (27.6)		20 (26.3)	47 (61.8)	9 (11.8)		26 (34.2)	49 (64.5)	1 (1.3)		37 (48.7)	34 (44.7)	5 (6.6)	
**Smoking**				0.049				0.364				0.668				0.921
- No	71 (21.5)	212 (64.2)	47 (14.2)		90 (27.3)	217 (65.8)	23 (7.0)		116 (35.2)	195 (59.1)	19 (5.8)		170 (51.5)	148 (44.8)	12 (3.6)	
- Quit	44 (21.2)	130 (62.5)	34 (16.3)		72 (34.4)	127 (60.8)	10 (4.8)		79 (37.8)	122 (58.4)	8 (3.8)		101 (48.3)	100 (47.8)	8 (3.8)	
- Yes	1 (10.0)	4 (40.0)	5 (50.0)		2 (20.0)	7 (70.0)	1 (10.0)		5 (50.0)	5 (50.0)	0 (0.0)		5 (50.0)	5 (50.0)	0 (0.0)	
**Skin itching disturbingly**				0.176				0.070				0.072				0.999
- No	92 (21.0)	284 (64.8)	62 (14.2)		127 (28.9)	290 (66.1)	22 (5.0)		161 (36.7)	252 (57.4)	26 (5.9)		222 (50.6)	201 (45.8)	16 (3.6)	
- Yes	25 (22.5)	63 (56.8)	23 (20.7)		37 (33.3)	63 (56.8)	11 (9.9)		39 (35.1)	71 (64.0)	1 (0.9)		56 (50.5)	51 (45.9)	4 (3.6)	
**Number of offspring#**, Mean (SD)	3.6 (2.4)	2.9 (1.9)	2.6 (1.0)	0.016	3.1 (2.2)	3.0 (2.0)	3.1 (1.3)	0.513	3.0 (1.8)	3.1 (2.1)	2.7 (2.4)	0.093	3.2 (2.3)	2.9 (1.7)	2.5 (1.4)	0.517

Data are presented as *N* (%) unless otherwise stated

N*; there may be some missing data i.e. some study subjects refused the use of data afterwards

^#^ = female participants only

### Predisposing and associative factors with benign skin tumors

#### Seborrheic keratosis

As shown in Table 1, patients with multiple site seborrheic keratosis were more likely to be male than female 21.6% and 12.1% cases, respectively (*p* < 0.05). Multiple tumors were also seen significantly more often in those with the history of outdoor working than in those without. Those with a darker skin type presented more often with multiple tumors than those with lighter skin. Similarly, among those who reported itching, multiple tumors were more common (20.7%) than in those without itching (14.2%) but this finding did not reach statistical significance. A significant association was found between smoking and multiple seborrheic keratosis. In females a lower number of offspring was significantly associated with a higher number of tumors.

In males, multiple seborrheic keratosis was seen more often in those with higher body mass index (BMI) when compared with those with lower BMI, and in those with higher fasting plasma glucose level (fP-gluc) than in those with lower fP-gluc, however, these findings did not reach statistical significance (Table 2).

**Table 2:** Benign skin tumors and their associations with anthropometric measurements and laboratory findings, stratified by sex. All laboratory parameters were taken as fasting samples. B: blood; P: plasma

	**Seborrheic keratosis**				**Lentigo solaris**				**Cherry angiomas**				**Melanocytic nevi**			
	**None**	**1–50**	** > 50**	***p* value**	**None**	**1–50**	** > 50**	***p* value**	**None**	**1–50**	** > 50**	***p* value**	**None**	**1–50**	** > 50**	***p* value**
N*	**117**	**347**	**86**		**164**	**353**	**34**		**201**	**323**	**27**		**278**	**253**	**20**	
**Females**																
*Age*	78.0 (4.9)	78.2 (4.3)	77.0 (3.7)	0.324	76.9 (4.6)	78.3 (4.3)	78.8 (3.7)	0.015	77.7 (3.9)	78.1 (4.7)	78.0 (3.7)	0.893	78.2 (4.6)	77.7 (4.2)	78.2 (4.0)	0.617
*Body mass index* (kg/m^2^)	28.0 (4.4)	27.5 (4.8)	26.9 (4.2)	0.343	28.3 (4.7)	27.3 (4.6)	27.6 (4.6)	0.464	27.8 (4.6)	27.6 (4.3)	25.0 (6.7)	0.190	27.3 (4.2)	27.7 (5.1)	27.7 (3.1)	0.479
*B- Haemoglobin* (g/l)	135.4 (12.2)	134.9 (10.6)	137.3 (11.1)	0.290	135.4 (10.9)	135.3 (11.0)	135.3 (12.9)	0.965	136.6 (11.5)	134.6 (11.1)	135.6 (8.1)	0.279	134.1 (10.5)	136.4 (11.4)	137.8 (14.5)	0.195
*P-glucose* (mmol/l)	5.8 (0.8)	5.9 (1.1)	5.9 (0.8)	0.764	6.2 (1.5)	5.8 (0.8)	6.0 (1.2)	0.268	6.0 (1.1)	5.8 (1.0)	5.6 (0.5)	0.151	5.9 (1.2)	5.8 (0.8)	5.8 (0.8)	0.908
*P- glycated haemoglobin* (mmol/mol)	40.3 (4.9)	39.9 (5.2)	40.9 (7.2)	0.607	40.5 (4.4)	39.8 (5.5)	41.5 (7.1)	0.202	41.4 (6.3)	39.6 (5.0)	38.8 (2.9)	0.031	40.5 (5.9)	39.7 (4.8)	40.4 (4.7)	0.640
*P-creatinine* (umol/l)	68.4 (19.5)	66.4 (16.5)	64.9 (13.4)	0.902	67.4 (21.4)	66.6 (15.5)	65.5 (15.4)	0.780	69.5 (19.5)	65.4 (15.6)	64.7 (14.0)	0.146	68.7 (18.4)	64.8 (15.2)	62.4 (14.7)	0.131
- *P- cholesterol (mmol/l)*	5.0 (1.1)	5.0 (1.1)	5.1 (1.3)	0.858	5.2 (1.1)	4.9 (1.1)	5.1 (1.0)	0.111	4.9 (1.1)	5.0 (1.1)	5.3 (1.2)	0.574	5.0 (1.1)	5.1 (1.1)	4.5 (1.4)	0.288
*P- high-density cholesterol* (mmol/l)	1.7 (0.4)	1.7 (0.4)	1.7 (0.5)	0.762	1.7 (0.4)	1.7 (0.4)	1.8 (0.5)	0.660	1.6 (0.4)	1.7 (0.4)	1.9 (0.4)	0.023	1.7 (0.5)	1.7 (0.4)	1.5 (0.5)	0.376
*P- low -density cholesterol*(mmol/l)	2.7 (0.9)	2.7 (0.9)	2.7 (1.0)	0.982	2.9 (0.9)	2.7 (0.9)	2.7 (0.9)	0.150	2.7 (0.9)	2.7 (0.9)	2.7 (0.9)	0.895	2.7 (0.9)	2.8 (0.9)	2.5 (1.2)	0.219
*P- triglycerides* (mmol/l)	1.3 (0.6)	1.2 (0.5)	1.3 (0.5)	0.511	1.4 (0.7)	1.2 (0.5)	1.3 (0.7)	0.283	1.4 (0.6)	1.2 (0.5)	1.2 (0.5)	0.002	1.3 (0.6)	1.2 (0.5)	1.2 (0.6)	0.828
**Males**																
*Age*	79.2 (3.7)	79.1 (3.9)	79.4 (3.3)	0.630	78.8 (3.9)	79.4 (3.5)	79.9 (3.8)	0.136	79.2 (3.6)	79.2 (3.8)	77.8 (3.8)	0.812	79.7 (3.9)	78.7 (3.4)	76.9 (3.5)	0.149
*Body mass index* (kg/m^2^)	25.8 (2.8)	27.2 (3.7)	27.1 (4.0)	0.267	27.0 (3.7)	26.8 (3.6)	27.2 (3.8)	0.857	27.1 (3.6)	26.8 (3.6)	25.8 (4.7)	0.433	26.8 (3.4)	27.2 (3.9)	24.9 (2.0)	0.221
*B-haemoglobin* (g/l)	140.3 (11.9)	143.0 (10.8)	143.9 (15.3)	0.211	142.2 (11.0)	143.5 (13.5)	142.2 (8.4)	0.543	141.6 (11.3)	144.1 (12.8)	132.2 (7.1)	0.041	141.9 (13.1)	143.4 (11.5)	148.0 (7.3)	0.448
*P- glucose* (mmol/l)	5.9 (0.9)	5.9 (0.9)	6.8 (2.8)	0.168	6.0 (1.0)	6.2 (2.0)	5.8 (0.6)	0.681	5.9 (1.2)	6.2 (1.8)	5.9 (1.3)	0.433	5.8 (0.7)	6.4 (2.1)	5.4 (0.4)	0.072
*P-glycated hemoglobin* (mmol/mol)	39.6 (6.9)	40.0 (7.0)	42.7 (10.5)	0.377	40.1 (7.5)	40.7 (8.4)	41.6 (7.6)	0.627	40.1 (8.1)	40.8 (7.9)	36.8 (5.1)	0.473	39.3 (5.8)	42.2 (9.6)	34.9 (3.6)	0.005
*P- creatinine* (umol/l)	85.1 (23.5)	86.2 (22.5)	85.2 (22.7)	0.662	85.7 (22.6)	86.4 (23.4)	80.6 (9.8)	0.865	86.7 (25.0)	84.2 (16.1)	113.2 (75.7)	0.950	86.7 (21.5)	85.6 (24.3)	76.3 (9.6)	0.330
*P-cholesterol* (mmol/l)	4.4 (1.1)	4.3 (1.0)	4.3 (1.2)	0.512	4.4 (1.1)	4.3 (1.0)	4.2 (0.7)	0.805	4.3 (1.0)	4.4 (1.1)	4.3 (1.1)	0.999	4.2 (1.0)	4.4 (1.1)	4.7 (1.3)	0.164
*P- high-density cholesterol* (mmol/l)	1.5 (0.4)	1.4 (0.4)	1.3 (0.3)	0.268	1.4 (0.4)	1.4 (0.4)	1.5 (0.3)	0.938	1.4 (0.4)	1.4 (0.4)	1.3 (0.5)	0.456	1.4 (0.4)	1.4 (0.4)	1.4 (0.3)	0.942
*P- low -density cholesterol* (mmol/l)	2.4 (0.9)	2.3 (0.8)	2.2 (1.0)	0.433	2.4 (0.9)	2.3 (0.9)	2.3 (0.7)	0.331	2.3 (0.8)	2.3 (0.9)	2.5 (1.0)	0.872	2.3 (0.9)	2.4 (0.8)	2.8 (1.0)	0.294
*P-triglycerides* (mmol/l)	1.1 (0.4)	1.2 (0.6)	1.4 (1.0)	0.375	1.2 (0.5)	1.3 (0.9)	1.0 (0.2)	0.642	1.3 (0.8)	1.2 (0.7)	1.1 (0.5)	0.975	1.1 (0.4)	1.3 (0.9)	1.1 (0.3)	0.608

N*; there may be some missing data i.e. some study subjects refused the use of data afterwards

All data are presented as mean (SD)

#### Lentigo solaris

Multiple-site lentigo solaris were seen significantly more often in females than in males. Those with a history of outdoor working were more likely to have multiple senile lentigos than those without, but the result did not reach statistical significance. In female participants, multiple-site lentigo solaris was significantly more common with advanced age (Table 1).

#### Cherry angiomas

Multiple cherry angiomas affected females significantly more often than males (Table 1). As shown in Table 2, a lower glycated haemoglobin (HbA1c) level was associated with a higher number of cherry angiomas in both sexes, reaching statistical significance in females (*p* < 0.05), but not in males (*p* = 0.473). Female subjects with multiple cherry angiomas had significantly higher high-density cholesterol (HDL) and lower triglyceride values than those with fewer cherry angiomas. In turn, lower mean haemoglobin (Hb) in males was significantly associated with the presence of multiple cherry angiomas. Lower BMI was weakly associated with the presence of multiple cherry angiomas in both sexes, but this finding did not reach significance in either males or females.

#### Melanocytic nevi

Individuals with no melanocytic nevi most commonly belonged to the skin type group I. In turn, those with multiple nevi more often than not had a history of outdoor working but this finding did not reach statistical significance. Those with multiple nevi seemed to have a smaller number of offspring but the evidence of this association was weak. Male participants with multiple melanocytic nevi were significantly more likely to have a lower HbA1c levels than those who had no nevi. A similar trend was seen in fP-gluc, but the result was not statistically significant.

## Discussion

This population-based study evaluated the etiological factors of benign skin tumors. We found several different predisposing factors, each of which could represent part of the etiologic process for these tumors. Furthermore, our findings confirm that benign skin tumors are exceedingly common in elderly people [7].

Almost 80% of our subjects had at least one benign skin tumor, and the presence of multiple tumors varied between tumor types, but affected as many as 15.6% of cases. The most common benign skin tumor was seborrheic keratosis, followed by lentigo solaris. This echoes the results of previous studies: an Iranian study of 259 persons aged 60 and over found benign neoplasms in 68.3% of the population, with seborrheic keratosis (49.4% participants) the most frequent [1]. A German study of 223 persons aged 65 and older found seborrheic keratosis 56% of the participants [2]. To date there is no exhaustive explanation as to why the frequency of benign tumors increases with age.

Despite their high incidence, the etiology of seborrheic keratoses has not been widely studied and remains partly unclear [23, 24]. We found seborrheic keratosis to affect a greater proportion of males than females. Previous studies have not found significant sex differences in the incidence of these lesions but in some studies males have been reported to have more seborrheic keratosis sites on the trunk and arms than females [24]. As mentioned previously, age is recognized as a risk factor for seborrheic keratosis: while it is not unknown in people as young as 15–25 years old, it can be present in 100% of populations over the age of 50 years [25]. When comparing the present results to the previous NFBC1966 study with younger participants (46-year-olds) the incidence of seborrheic keratosis was clearly higher in the present study than among the younger cohort members both in females (76.6% vs. 47.2%) and males (82.4% vs. 40.9%) [8]. Previous studies have also found some evidence of a role of UV radiation in the pathogenesis of seborrheic keratosis [14, 24]. We similarly found that a history of outdoor working was associated with a higher number of seborrheic keratosis sites. Smoking ang pregnancy have also been proposed as risk factors for seborrheic keratosis [26, 27]. A Chinese study found that smoking was associated with seborrheic keratosis [26]. We found multiple lesions to be more common among smokers than in those never smoked. In general pregnancy is known to alter the frequency of skin tumors, specifically increasing the likelihood of seborrheic keratosis [27]. Interestingly, we found that females with multiple seborrheic keratosis tended to report a lower number of offspring. However, lack of evidence prevents the confirmation of any relationship between seborrheic keratosis and pregnancy. We also found that those with multiple seborrheic keratosis reported more itching than those without (although the result was not statistically significant). Itching is an uncomfortable sensation that leads to scratching and has been linked to multiple psychosocial symptoms such as depression and anxiety [28]. Furthermore, our male participants with multiple lesions tended to have higher BMI and higher fP-gluc levels when compared with those with fewer lesions. Nevertheless, more studies are needed to confirm any association between metabolism and seborrheic keratosis.

We found a female predominance in numbers of cases of lentigo solaris, a finding that differed from that of a Turkish study in which lentigo solaris was more common in males than females (83.9% vs 77.3%) [29]. This difference may at least partially be explained by ethnicity, since our population was entirely of Caucasian origin. However, we did not find any significant differences between skin types. Lentigo solaris is well known to be strongly determined by prolonged exposure to UV radiation, but air pollution may play a role as well [12, 13]. In our study, cases of lentigo solaris were also more frequently seen among the subjects with a history of outdoor working than in those without, but the result did not reach statistical significance. The prevalence of lentigo solaris lesions was a markedly higher in this older study population than in the 46-year-old subjects of the previous NFBC1966 study, both in males (56.6% vs. 10.1%) and females (78.3% vs. 16.6%) [8].

The pathology of cherry angiomas (haemangioma, vascular lesion) is not completely known and there are only a few studies of their risk factors [10, 11]. In our study, cherry angiomas were more common among females, nevertheless, previous studies have recorded contradictory findings on sex differences [8, 30]. Some somatic gene mutations and pregnancy have been reported to as risk factors for cherry angiomas [1, 11], but the role of hormonal factors in the pathogenesis of cherry angiomas is still unclear [31]. Interestingly, in our study, cherry angiomas were more common in females with fewer children. However, the evidence for an association was weak and this postulate needs to be studied in larger populations. We noticed also a trend towards a ‘more healthy metabolism’ among subjects with multiple cherry angiomas: these subjects had lower HbA1c and triglyceride levels and higher HDL levels. There were no statistically significant associations with total cholesterol or low-density cholesterol (LDL), but a lower BMI was associated with the presence of multiple cherry angiomas in both female and male participants. Interestingly, male subjects with multiple cherry angiomas had also lower hemoglobin values. The incidence of cherry angiomas increases with age as do those of seborrheic keratosis and lentigo solaris, however, the increase was not so distinct with cherry angiomas [10, 11]: the incidence of cherry angiomas in the NFBC1966 study of 46-year-olds the incidence was only a little lower than in the present study both in female (67.0% vs. 65.9%) and male (57.6% vs. 54.2%) [8].

### Melanocytic nevi

Fitzpatrick skin type affected the incidence of melanocytic nevi, although there was a great deal of variation in skin group sizes. In general, people with a lighter skin type are at greater risk of skin damage caused by the sun and getting both melanoma and non-melanoma skin cancers [32]. The largest proportion of participants with no nevi was found in the skin type I group, and the type IV group had the highest proportion with multiple nevi. This contradicts the findings of some previous studies, which reported that lighter skin carries a greater risk for melanocytic nevi, however, those studies mostly had a population far younger than ours [33–35].There was also an association between outdoor working and multiple melanocytic nevi – confirming that the cumulative amount of UV radiation increases the number of nevi [15]. Interestingly, a higher number of melanocytic nevi was associated with better glucose metabolism (lower HbA1c and fP- gluc levels), and to the best of our knowledge, our study is the first to find such an association. Previous studies have found various other factors to be associated with the onset of melanocytic nevi; for example immunosuppression and immunodeficiency correlate with higher numbers of nevi, and genetic factors also play a role [15]. In addition, hormonal factors (such as pregnancy and puberty) have been shown to affect the development of nevi [15]. However, among our female participants we found no significant association between number of offspring and the presence of melanocytic nevi, although there were signs of a trend towards multiple nevi in participants with fewer offspring.

To date our TBSE study is the one of the largest in an elderly general population. The fact that the skin evaluation was performed by experienced dermatologists decreases the risk of bias that might result from erroneous diagnoses. The unique design of the birth cohort allowed us comprehensively to investigate the etiology of these lesions and to examine multiple metabolic measurements as candidate risk factors. One limitation of the study was that not all invited subjects chose to participate, but considering the relatively high age of the study population, the participation rate in the clinical examination was moderately good. Furthermore, some data were missing from the health questionnaires and some statistical analyses may have been underpowered due to the rather small study population. While we assessed the effect of a history of working outdoors, leisure time spent outdoors was not accounted for, which may have affected the results. The ethnic homogeneity of our population means that our results cannot be generalized beyond other similarly-aged Caucasian populations.

In conclusion, the prevalence of benign skin tumors increases with advanced age. As a result, it can be more challenging to differentiate malignant skin changes from benign ones [7, 36]. For example, clinically distinguishing melanoma from seborrheic keratosis can sometimes be difficult [4, 37]. Benign skin tumors easily cause distress and discomfort, and are one of the most common reasons for a physician visit, [9] thus contributing to the overall burden on healthcare services. We found some interesting possible factors associating with benign skin tumors such as smoking with seborrheic keratosis and the number of offspring in females with cherry angiomas. However, more research is needed to strengthen the clinical relevance of our findings and to add knowledge of the etiology and pathogenesis of benign skin lesions, which could in turn lead to the enhancement of prevention strategies.

## Supplementary Information

Supplementary Material 1.

## Data Availability

The data that support the findings of this study are available from Northern Finland Birth Cohort 1966 Study. Restrictions apply to the availability of these data, which were used under license for this study. The use of personal data is based on cohort participant’s written informed consent at his/her latest follow-up study, which may cause limitations to its use. Permission to use the data can be applied for research purposes via electronic material request portal. Please, contact NFBC project center (NFBCprojectcenter@oulu.fi) and visit the cohort website (www.oulu.fi/nfbc) for more information.

## Abbreviations

UV: Ultra-violet
NFBC1966: Northern Finland Birth Cohort 1966
TBSE: Total body skin examination
ANOVA: Analysis of variance
BMI: Body mass index
