# Supplementary material for: Benign skin tumors in older persons: a population-based study
Source: BMC Geriatr. 2025 Apr 5;25:226. doi: 10.1186/s12877-025-05881-1 (PMC11971830; doi:10.1186/s12877-025-05881-1)
Supplement: Supplementary file 1 — Supplementary Material 1. [file 12877_2025_5881_MOESM1_ESM.docx]

**Appendix 1**

**Supplement Benign Skin Tumors**

**Anthropometric measurements**

All clinical health examinations took place and all measurements were taken during the Parents Study. The examination included body weight in light clothing which was measured with a digital scale (calibrated regularly). Height was measured twice using a standard and calibrated stadiometer and the mean of the two measurements was used. Body mass index (BMI) was calculated as the ratio of weight to height squared (kg/m^2^). Waist and hip circumferences were measured twice (mean of the two measurements was used) and the waist–hip ratio was assessed as the ratio between circumferences of the waist (at the level midway between lowest rib margin and the iliac crest) and the hip (at the widest trochanters). All measurements were done after overnight (12 h) fasting period.

**Fasting plasma glucose and glycated haemoglobin**

Venous blood samples were used to determine fasting plasma glucose and glycated haemoglobin (HbA1c) –values of every eligible participant. Blood samples were taken at the cohort laboratory between 7:00 and 11:00 a.m. after an over-night (12h) fast. The concentration of HbA1c and the concentration of total haemoglobin were measured by immunochemical assay method. Glucose were analysed using an enzymatic hexokinase/glucose-6-phosphate dehydrogenase method. (both method: Advia 1800; Siemens Healthcare Diagnostics Inc.. Tarrytown. Ny. USA). The samples were analysed in NordLab Oulu. a testing laboratory (T113) accredited by Finnish Accreditation Service (FINAS) (EN ISO 15189).

**Other biochemical measurements**

Blood samples were taken after an overnight fasting period, centrifuged immediately and stored firstly at −20°C and later at −80°C. All blood samples were analysed in the laboratory of the University Hospital of Oulu according to a standardized protocol. Serum total cholesterol, high-density lipoprotein (HDL), low-density lipoprotein (LDL), triglycerides and creatine were determined using an enzymatic assay method (Advia 1800; Siemens Healthcare Diagnostics Inc.. Tarrytown. NY. USA).

**References**

1. Shanewise. J. S. *et al*. ASE/SCA guidelines for performing a comprehensive intraoperative multiplane transesophageal echocardiography examination: recommendations of the American Society of Echocardiography Council for Intraoperative Echocardiography and the Society of Cardiovascular Anesthesiologists Task Force for Certification in Perioperative Transesophageal Echocardiography. *J. Am. Soc. Echocardiogr.* **12**. 884-900 (1999).

2. Parikh. N. I. *et al*. A risk score for predicting near-term incidence of hypertension: the Framingham Heart Study. *Ann. Intern. Med.* **148**. 102-110 (2008).

3. Conroy. R. *et al*. Estimation of ten-year risk of fatal cardiovascular disease in Europe: the SCORE project. *Eur. Heart J.* **24**. 987-1003 (2003).

4. Bhopal. R. *et al*. Predicted and observed cardiovascular disease in South Asians: application of FINRISK. Framingham and SCORE models to Newcastle Heart Project data. *Journal of public health* **27**. 93-100 (2005).

5. Vartiainen. E.. Laatikainen. T.. Peltonen. M. & Puska. P. Predicting coronary heart disease and stroke: The FINRISK Calculator. *Global heart* **11**. 213-216 (2016).

6. Bedogni. G. *et al*. The Fatty Liver Index: a simple and accurate predictor of hepatic steatosis in the general population. *BMC gastroenterology* **6**. 33 (2006).
